# Spatiotemporal expression of *IgLON* family members in the developing mouse nervous system

**DOI:** 10.1038/s41598-021-97768-5

**Published:** 2021-10-01

**Authors:** Sydney Fearnley, Reesha Raja, Jean-François Cloutier

**Affiliations:** 1 The Neuro, Montreal Neurological Institute - Hospital, 3801 University, Montréal, QC H3A 2B4 Canada; 2 Department of Neurology and Neurosurgery, McGill University, Montréal, Canada; 3 Department of Anatomy and Cell Biology, McGill University, Montréal, Canada

**Keywords:** Cellular neuroscience, Development of the nervous system, Neural circuits, Olfactory system, Peripheral nervous system, Developmental biology, Neuroscience

## Abstract

Differential expression of cell adhesion molecules in neuronal populations is one of the many mechanisms promoting the formation of functional neural circuits in the developing nervous system. The IgLON family consists of five cell surface immunoglobulin proteins that have been associated with various developmental disorders, such as autism spectrum disorder, schizophrenia, and major depressive disorder. However, there is still limited and fragmented information about their patterns of expression in certain regions of the developing nervous system and how their expression contributes to their function. Utilizing an in situ hybridization approach, we have analyzed the spatiotemporal expression of all *IgLON* family members in the developing mouse brain, spinal cord, eye, olfactory epithelium, and vomeronasal organ. At one prenatal (E16) and two postnatal (P0 and P15) ages, we show that each *IgLON* displays distinct expression patterns in the olfactory system, cerebral cortex, midbrain, cerebellum, spinal cord, and eye, indicating that they likely contribute to the wiring of specific neuronal circuitry. These analyses will inform future functional studies aimed at identifying additional roles for these proteins in nervous system development.

## Introduction

The developing nervous system relies on many mechanisms to form functioning neural circuits. One such mechanism is cell–cell or axon–axon interaction which relies on adhesion between cell surface molecules belonging to the large family of cell adhesion molecules (CAMs). By allowing for adhesive interactions, CAMs modulate a wide array of processes during neuronal development, including neurogenesis, fasciculation and guidance of axons, and synapse formation and selectivity^1–4^. The selective expression of different families of CAMs, or of individual members of specific subfamilies, in populations of neurons is especially important during the development of sensory neural maps. For example, differential expression of members of the Teneurin and Kirrel family of CAMs have been implicated in the formation of the olfactory maps in *Drosophila* and mice, respectively^5–10^.

The IgLON family of cell surface proteins belongs to the large immunoglobulin family of CAMs. To date, five *IgLON* family members have been identified: opioid-binding cell adhesion molecule (OBCAM/IgLON1), neurotrimin (Ntm/IgLON2), limbic-system associated membrane protein (LAMP/LSAMP/IgLON3), neuronal growth regulator 1 (NEGR1/Kilon/IgLON4), and IgLON5^11–14^. IgLONs are composed of three C2-like Ig domains and are tethered to the surface of the membrane by a glycosylphosphatidylinositol anchor^15^. Each Ig domain can be glycosylated, but glycosylation sites vary between IgLON family members^16^. The organization of *IgLON* genes also varies across family members. While a dual-promoter structure has been identified for *IgLON1-3* genes, leading to expression of two different isoforms, a single promoter regulates the expression of *IgLON4* and *IgLON5*^17–21^ On the extracellular surface of the plasma membrane, IgLONs form homo- and heterodimers that facilitate adhesion^22–26^. *IgLONs* have been implicated in the regulation of several neurodevelopmental processes including neurite outgrowth, axonal fasciculation, and synaptogenesis^27,28^. IgLON4 can promote neurite outgrowth and attract hippocampal neuron axons, and its ablation reduces hippocampal neurite outgrowth^29,30^ Hippocampal neurons deficient for IgLON2 showed premature sprouting and increased elongation, suggesting IgLON2 may have an inhibitory role, whereas IgLON3 likely promotes neurite initiation^31^. Conversely, an inhibitory effect of IgLON3 on neurite outgrowth was observed in dorsal root ganglia neurons^32^. Proper fasciculation of dopaminergic afferent fibers and guidance to the lateral habenula has been shown to require IgLON3 expression on lateral habenula efferent projections^33,34^. Furthermore, shedding of IgLONs from the cell surface by metalloproteinases promotes neurite outgrowth in both cortical and dorsal root ganglion neurons^35,36^. Evidence for a role of IgLONs in modulating synapse development include the observation that expression of IgLON1 or IgLON3 in rat hippocampal neuron cultures leads to increased synapse numbers, while overexpression of IgLON4 reduced synapse numbers^37^. While IgLONs are conserved across deuterostomes, protostomes such as *Drosophila* have evolved a pair of binding partners called DIPs and DPRs, whose combinatorial expression contributes to the sorting and targeting of olfactory receptor neuron axons^38^.

*IgLONs* have been linked to various developmental disorders. Mice with an *IgLON2* deletion show decreased performance in the active avoidance task, suggesting a deficit in emotional learning^39^. Polymorphisms in the *IGLON3* gene have been associated with major depressive disorder, panic disorder, and schizophrenia^40–42^. Interestingly, mice bearing a deletion in the *IgLON3* gene showed reduced anxiety in novel environments and lowered sensitivity to stressors^43^. Genome-wide association studies have linked *IGLON4* with increased risk of major depressive disorder^44^. As well, placental DNA methylation studies associated *IGLON4* with increased body mass index (BMI) and neurodevelopment defects in children^45^. Mouse knock-out models of *IgLON4* showed increased adiposity and behavioral deficits associated with neuropsychiatric disorders^46–48^. IgLON5, the least characterized family member, was first identified in patients with anti-IgLON5 disorder that present a variety of symptoms, including altered sleep, gait abnormalities, bulbar dysfunction, and chorea^13,49,50^.

Despite *IgLONs*’ roles in key neurodevelopmental events, there is fragmented information available in the literature about their expression in certain regions of the developing nervous system^17,21,22,32,51–53^. We utilized an in situ hybridization approach to create a spatiotemporal map of *IgLON* mRNA expression in the murine nervous system. We have analyzed one prenatal and two postnatal ages for the expression of the five *IgLON* family members. We demonstrate that during late prenatal and postnatal development, *IgLONs* are differentially expressed in specific regions of the nervous system, including the olfactory system and the developing eye. Our study provides data to inform future functional studies into the role of these adhesion molecules in the development of specific structures of the nervous system.

## Results

### Specificity of the cRNA probes

**Figure 1:**
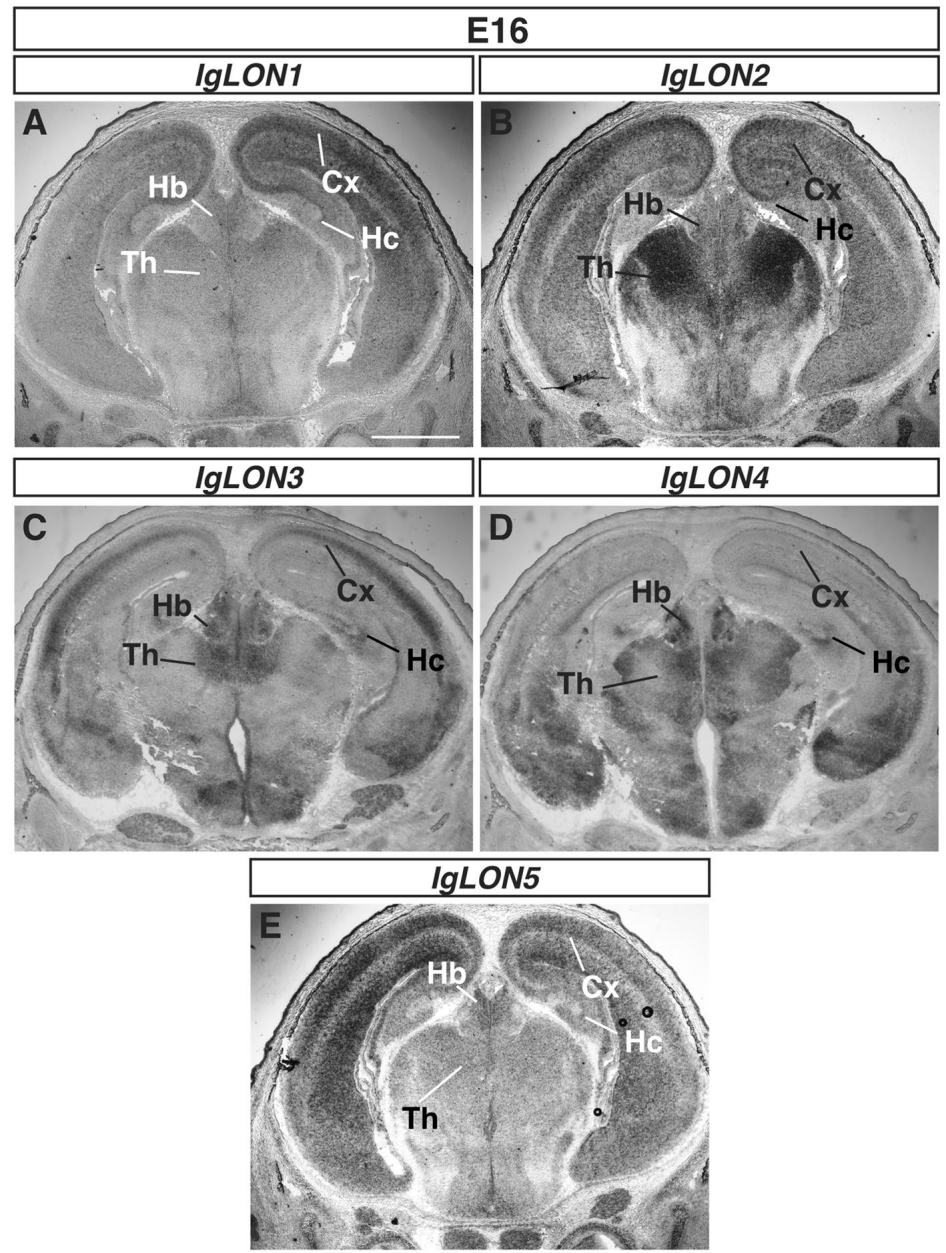
mRNA expression of *IgLON* family members in the developing brain. In situ hybridization of coronal sections of brains from E16 embryos with antisense cRNA probes specific for *IgLON1* (**A**), *IgLON2* (**B**), *IgLON3* (**C**), *IgLON4* (**D**), and *IgLON5* (**E**) transcripts. *IgLON1–5* are widely expressed in the brain but show differential expression in certain regions, such as the cortex and thalamus. *IgLON-1*, *2*, *3* and *5*, are expressed in the developing cortex (Cx), while *IgLON1–5* are expressed in the habenula (Hb) and various nuclei of thalamus (Th) (**A**, **B**, **C**, **D**, **E**). Scale bar = 1 mm.

**Figure 2:**
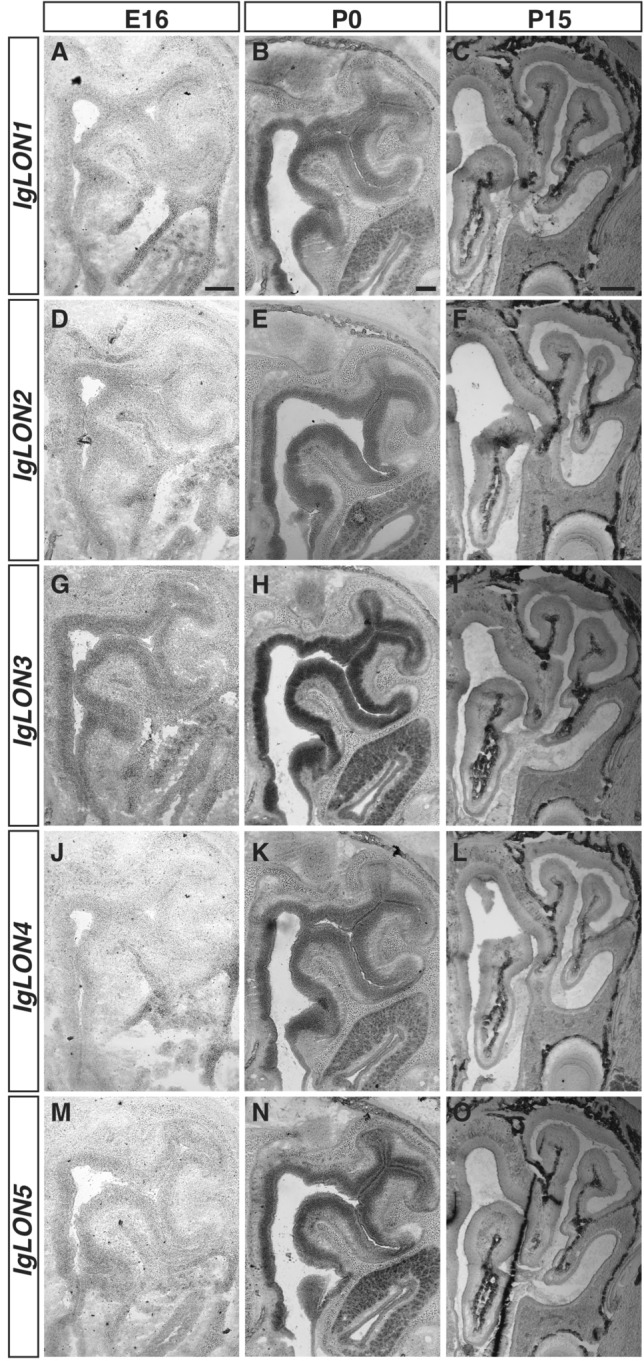
*IgLONs* mRNA expression in the developing olfactory epithelium (OE). In situ hybridization of coronal sections of OE from E16, P0, and 15 mice with antisense cRNA probes for *IgLON* transcripts. *IgLON3* mRNA is detected in the OE at E16 (**G**), while other *IgLON* family members could not be detected in the OE at that age (**A**, **D**, **J**, **M**). By P0 all *IgLONs* are expressed throughout the OE (**B**, **E**, **H**, **K**, **N**) but their expression is downregulated by P15 (**C**, **F**, **I**, **L**, **O**). Scale bars = 200 µm (**A**, **B**, **D**, **E**, **G**, **H**, **J**, **K**, **M**, **N**) and 500 µm (**C**, **F**, **I**, **L**, **O**).

**Figure 3:**
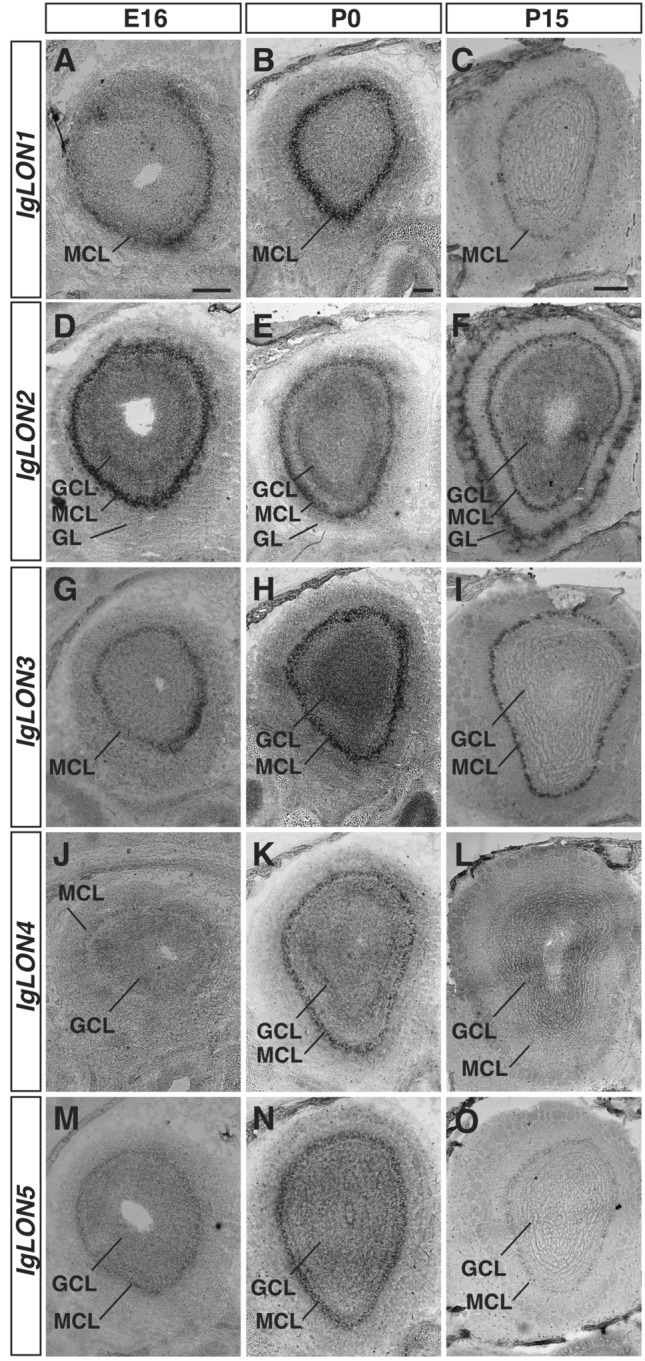
*IgLONs* mRNA expression in the developing olfactory bulb (OB). In situ hybridization of coronal sections of OB from embryonic day (E) 16 and postnatal day (P) 0 and 15 mice with antisense cRNA probes for *IgLON* transcripts. *IgLON1* mRNA is detected in the mitral cell layer (MCL) of the OB at E16 and is maintained at P0 and P15 (**A**–**C**). *IgLON2* mRNA expression is observed in the MCL and in the granule cell layer (GCL) of the OB at E16, P0, and P15 (**D**–**F**). By P15, *IgLON2* expression is also detected in the glomerular cell layer (GL) (**F**). *IgLON3* is expressed in the MCL at E16, P0, and P15, and could also be detected in the GCL at P0 (**G**–**I**). *IgLON4* mRNA expression is observed at low levels in the GCL and in the MCL at E16 (**J**). Its expression in these regions is increased at P0 but is downregulated in the MCL at P15 (**K**, **L**). *IgLON5* mRNA is detected in the MCL and GCL at E16 (**E**) and P0 (**N**) and at lower levels at P15 (**O**). Scale bars = 200 µm (**A**, **B**, **D**, **E**, **G**, **H**, **J**, **K**, **M**, **N**) and 500 µm (**C**, **F**, **I**, **L**, **O**).

**Figure 4:**
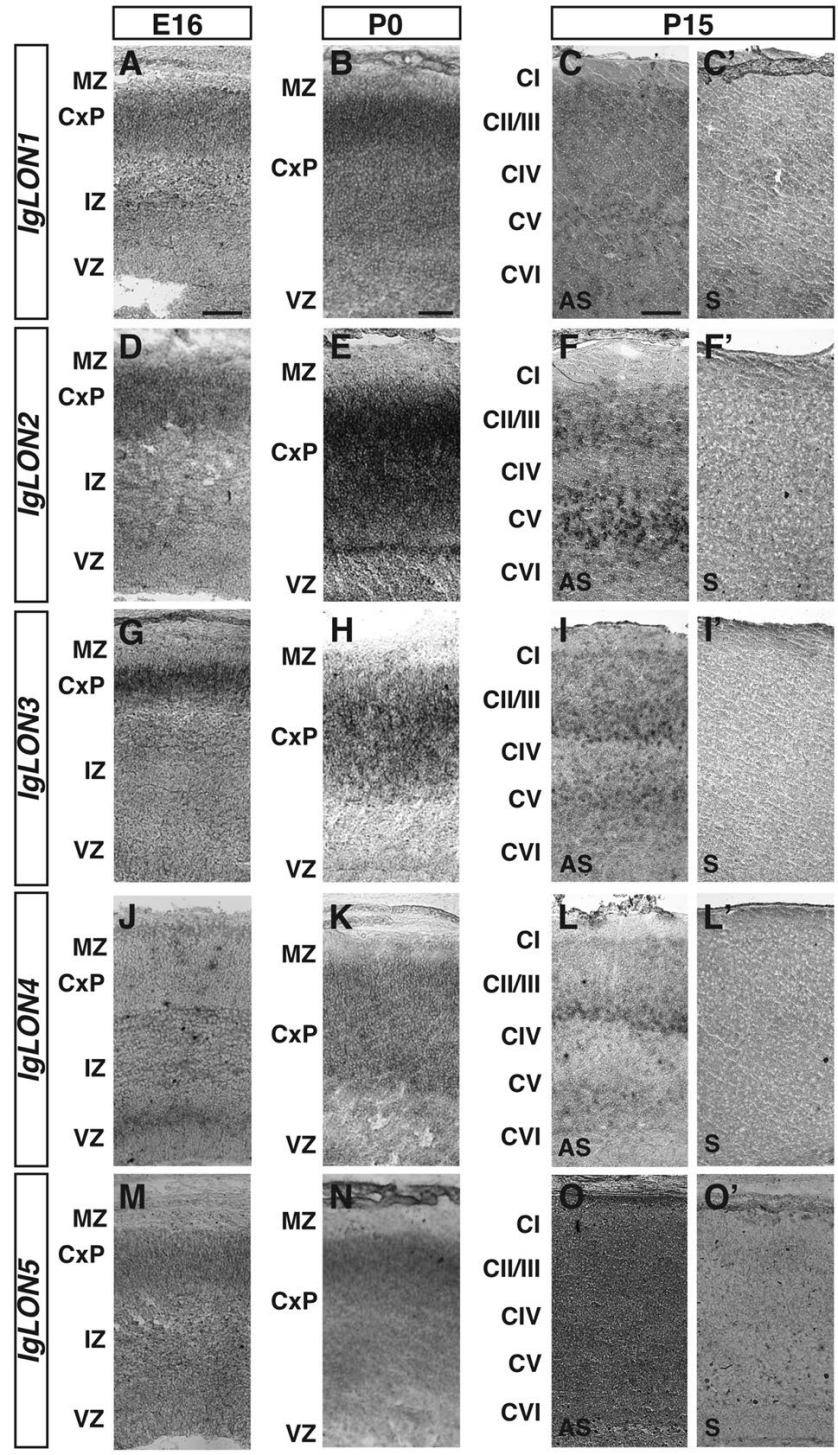
*IgLONs* mRNA expression during development of the cerebral cortex. In situ hybridization of coronal sections of cerebral cortex from E16, P0, and P15 mice with antisense (**A**–**O**) or sense (**C′**–**O′**) cRNA probes for *IgLON* genes. *IgLON1* was expressed in the cortical plate (CxP) at E16 and P0 (**A**, **B**). At P15, *IgLON1* mRNA was observed in cortical layers II/III (CII/CIII) and V–VI (CV–VI) (**C**). *IgLON2* mRNA was detected in the CxP at E16 and upregulated in the CxP at P0 (**D**, **E**). At P15, *IgLON2* is expressed in layers CII/III and CV–VI of the cortex (**F**). *IgLON3* expression was detected in the CxP at E16 (**G**) and P0 (**H**), and in layers CII/III and CV-VI at P15 (**I**). *IgLON4* mRNA expression is restricted to the apical region of the ventricular zone (VZ) at E16 and at lower levels in the intermediate zone (IZ) (**J**). At P0, *IgLON4* expression was detected in the CxP (**K**). Expression at P15 was observed in layers CII/III and CV–VI (**L**). *IgLON5* was expressed at low levels in the CxP at E16 and P0 (**M**, **N**). At P15, *IgLON5* mRNA was observed at low levels throughout the cortex (**O**). Scale bars = 125 µm (**A**, **D**, **G**, **J**, **M**), 250 µm (**B**, **E**, **H**, **K**, **N**), and 500 µm (**C**, **C′**, **F**, **F′**, **I**, **I′**, **L**, **L′**, **O**, **O′**).

To examine the patterns of expression of *IgLON* family members in the developing nervous system, we incubated sections of embryonic day (E) 16 embryos, postnatal day (P) 0, and P15 brains with sense and antisense cRNA probes for *IgLON* mRNAs. cRNA probes were designed to detect expression of both the 1a and 1b transcripts for *IgLON1*, *IgLON2*, and *IgLON3*. Representative sections showing the specific patterns of expression of *IgLON1–5* are shown in Fig. 1, and no signal was observed on sections incubated with sense probes throughout our studies (as exemplified in Figs. 4, 6, and 8). To assess the potential cross-reactivity of the cRNA probes among *IgLON* family members, we reasoned that the observation of a region detected by a single cRNA probe would provide evidence that cRNA probes for other family members do not cross-react with this family member. For three of the family members, *IgLON2*, *IgLON3*, and *IgLON4*, we were able to detect specific structures that were positive for a single *IgLON* family cRNA probe. For example, the glomerular cell layer of the olfactory bulb (Fig. 3F) only expressed *IgLON2* at P15, the olfactory epithelium only expressed *IgLON3* at E16 (Fig. 2G), and the apical ventricular zone of the cortex was only positive for *IgLON4* at E16 (Fig. 4J). Cross-reactivity was also assessed by identifying specific brain regions that were positive for one *IgLON* and negative for another. For example, the expression of *IgLON2, 3,* and *5* in the eye, and of *IgLON4* in the entorhinal cortex, was not detected with the *IgLON1* cRNA probe, indicating that it does not bind to these mRNAs (Figs. S2D and S3B, C, E). Overall, we were able to find specific examples of brain areas that were positive for one *IgLON* and negative for another to cover most combinations of possible cross-reactivity between the probes as summarized in Table 1, demonstrating the specificity of the cRNA probes used.

**Table 1:** Summary of *IgLON* gene expression in the developing murine nervous system.

*IgLON*	E16					P0					P15				
	1	2	3	4	5	1	2	3	4	5	1	2	3	4	5
**I. Telencephalon**															
***Eye***															
Neuroblastic layer	−	+ +	+ + +	−	−										
Inner granular layer	−	+ +	+ + +	−	+ + +										
***Olfactory system***															
Vomeronasal organ	−	+	+ +	−	+ +	+ + +	+ + +	+ + +	−	+ + +	−	−	−	−	−
Olfactory epithelium	−	−	+ +	−	−	+ +	+ +	+ + +	+ +	+ +	−	−	−	−	−
Glomerular layer	−	−	−	−	−	−	+	−	−	−	−	+ + +	−	−	−
Mitral cell layer	+ + +	+ + +	+ + +	−	+ +	+ + +	+ + +	+ + +	+ +	+ +	+ +	+ + +	+ + +	−	+
Granular layer	−	+ +	−	−	−	−	+ +	+ + +	+	+	−	+ +	−	+ +	+
***Cerebral cortex***															
Cortical plate	+ +	+ + +	+ + +	−	+ +	+ +	+ + +	+ + +	+ +	+ +					
Intermediate zone	+	−	−	+	−										
Ventricular zone	−	−	−	+ +	−	−	−	−	−	−					
CI											−	−	−	−	+
CII–CIII											+ +	+ +	+ + +	+ +	+
CIV–CVI											+	+ + +	+ + +	+	+
***Entorhinal cortex***	−	+	+ + +	+ + +	+										
*** Hippocampal formation***	+ +	+ +	+ +	+ +	+										
CA1						+ + +	+ +	+ + +	−	+ +	+ +	+ + +	+ + +	−	+ +
CA3						+ + +	−	+ + +	+ +	+ +	+ + +	−	+ + +	+	+ +
Dentate gyrus						+	−	+ +	+ + +	+	+ +	−	+ + +	+ + +	+
**II. Diencephalon**															
***Thalamus***	+ +	+ + +	+ +	+ + +	+	+ +	+ + +	+ +	+	+	+	+ +	+ +	+	+
***Habenula***	+ +	+ +	+ +	+ + +	+	+ +	+ +	+ +	+	+	+ +	+ +	+ +	+	+
**III. Rhombencephalon**															
***Cerebellum***															
Molecular layer						−	−	−	−	−	−	−	−	−	−
Purkinje layer						+	+ + +	+	−	+	+ +	+ + +	+	−	+
Inner granular layer						−	+	+ +	−	−	−	+	+ +	+ +	+ +
**IV. Other**															
***Spinal cord***						+	+ + +	+ + +	+ +	+ +	+	+ + +	+ + +	+ +	+
***Dorsal root ganglion***						+	+ + +	+ + +	+	+ +	+	+ + +	+ + +	+	+

Relative gene expression: − , not detected; + , low expression; +  + , medium expression; +  +  + , high expression, E, embryonic; P, postnatal day.

### *IgLON* expression in the olfactory systems

Olfactory sensory neurons (OSNs) located in the olfactory epithelium (OE) are responsible for detecting odorants. OSNs project their axons to the olfactory bulb (OB) where they coalesce into neuropil structures termed glomeruli. In these structures, OSN axons form synapses with second order neurons, the mitral/tufted cells that send projections to pyramidal cells in the olfactory cortex. In contrast, vomeronasal sensory neurons (VSNs) located in the vomeronasal organ detect chemosignals that modulate social and sexual behaviour in mice and project their axons to the accessory olfactory bulb^54^. We examined the expression of *IgLONs* in the olfactory systems at E16, when OSN and VSN axons are projecting to their targets, P0 when glomeruli start to form, and P15 when glomeruli are refined^55,56^. While at E16, we could only detect *IgLON3* (Fig. 2A, D, G, J, M) in the OE, all *IgLON* family members were expressed uniformly throughout the OE by P0 (Fig. 2B, E, H, K, N). Interestingly, the levels of *IgLON* family members in the OE appear to decrease beyond detectable levels by P15 (Fig. 2C, F, I, L, O). In the OB (Fig. 3), *IgLON1*, *IgLON3*, and *IgLON5* mRNA were mainly detected in the mitral cell layer (MCL), with *IgLON3* mRNA also in the granule cell layer (GCL), which contains cells implicated in lateral inhibition of the mitral cells. While *IgLON1* and *IgLON3* MCL expression was maintained from E16 to P15 (Fig. 3A–C, G–I respectively), *IgLON5* expression decreased by P15 (Fig. 3M–O). *IgLON2* mRNA was detected in the MCL and GCL at all ages examined (Fig. 3D–F). It was also expressed in periglomerular cells present in the glomerular layer (GL) at P15 (Fig. 3F). *IgLON4* was detected in the MCL and GCL at E16 and P0, but only in the GCL at P15 (Fig. 3J–L). As observed in the OE, most *IgLONs* are expressed in the vomeronasal organ (VNO) by P0, except for *IgLON4*, and their expression decreases by post-natal day 15 (Fig. S1A–O). At E16, *IgLON3* and *IgLON5* mRNA were detected more basally in the VNO, but both genes were expressed throughout the VNO by P0 (Fig. S1G, H, M, N). *IgLON1* and *IgLON2* also showed uniform expression in the VNO at P0 but could not be detected at P15 (Fig. S1B, E, C, F).

### *IgLON* expression in the cortex

Cells from the ventricular zone and ganglionic eminences migrate during embryogenesis towards the cortical plate and develop into cortical neurons and interneurons that form the layered cortex. By E16, proliferative cells in the ventricular zone (VZ) have begun to migrate out to the cortical plate (CxP). By birth, cortical layer formation starts to emerge and further development leads to the fully formed cortex containing six neuronal layers. These layers are composed of neurons with different morphological features that serve various functions and that project their axons to a wide variety of targets, including the thalamus and the contralateral cortex^57–59^. Several *IgLONs* are expressed in the developing somatosensory cortex and some of their expression is maintained postnatally. At E16, *IgLON1*, *IgLON2*, *IgLON*3, and *IgLON5* were detected in the cortical plate (CxP). While *IgLON1, IgLON2*, and *IgLON5* were expressed throughout the CxP, *IgLON3* expression was higher in the apical region of the CxP (Fig. 4A, D, G, M). In contrast, *IgLON4* expression was mainly observed in the ventricular zone (VZ), where dividing cells are located, as well as at low levels in the intermediate zone^60^ (Fig. 4J). The expression of several *IgLON* family members was maintained as development proceeds into early post-natal stages. At P0, all family members were detected in the cortical plate. While *IgLON1* expression appeared higher in the apical region of the CxP, *IgLON2*, *IgLON3*, *IgLON4,* and *IgLON5* were detected throughout the CxP (Fig. 4B, E, H, K, N). At P15, all *IgLON* family members could be detected in the cortex (Fig. 4C, F, I, L, O). *IgLON1* was weakly detected in cortical layers II-III (CII-CIII) and V-VI (CV-VI), while *IgLON2*, *IgLON3,* and *IgLON4* mRNA were detected at high levels in layers CII-CIII and CV-VI, and *IgLON5* showed low levels of expression throughout the cortex (Fig. 4C, F, I, L, O).

### *IgLON* expression in the diencephalon

**Figure 5:**
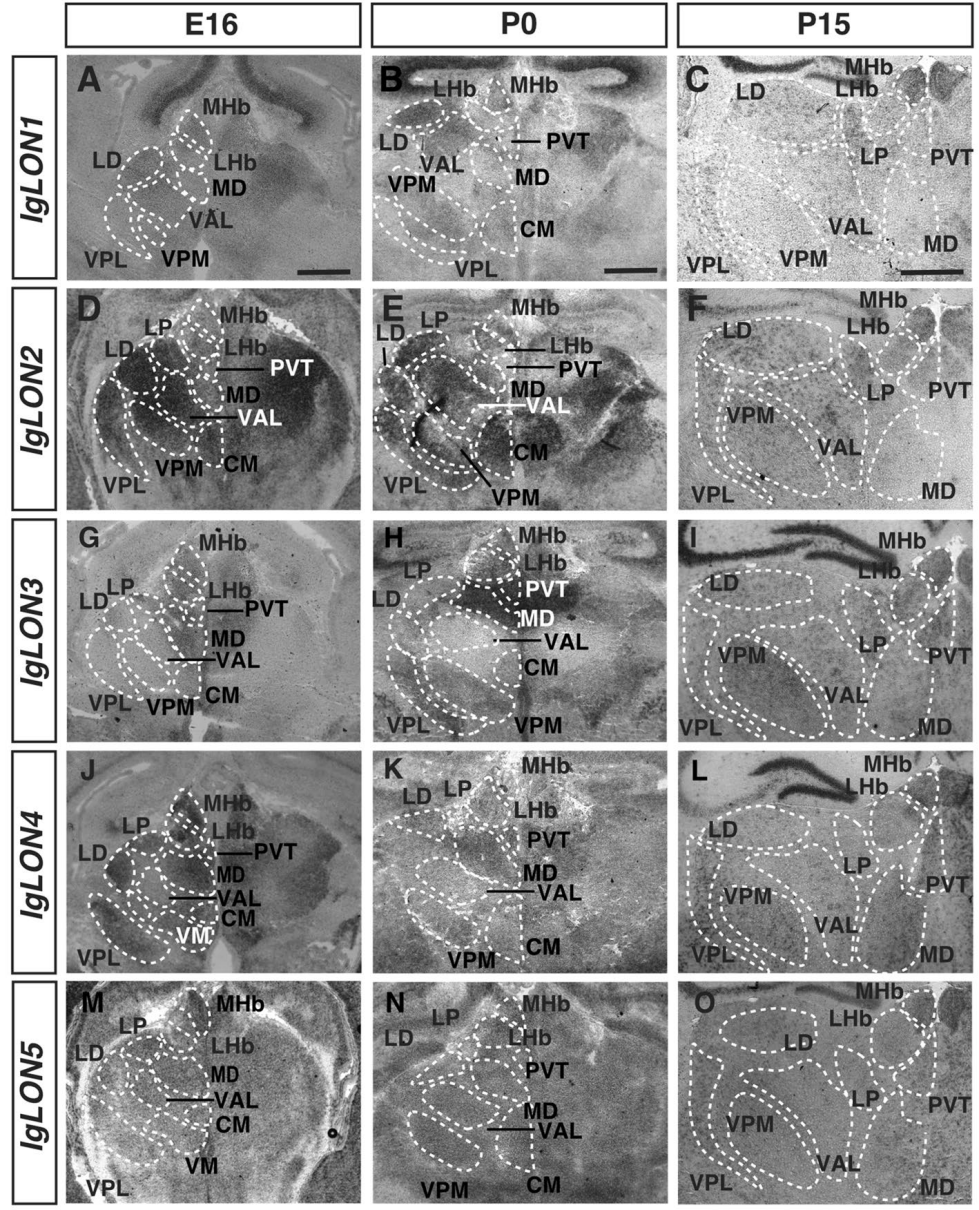
*IgLONs* mRNA expression in the developing thalamus. In situ hybridization of coronal sections of the thalamus from E16, P0, and 15 mice with antisense cRNA probes for *IgLON* transcripts. *IgLON1* mRNA was detected in the medial habenula (MHb), laterodorsal thalamic nucleus (LD), and ventral anterolateral thalamic nucleus (VAL) at all ages examined. *IgLON1* mRNA was also detected in additional nuclei, such as the ventral posteromedial thalamic nucleus (VPM) and ventral posterolateral thalamic nucleus (VPL) at P0 and P15, respectively (**A**–**C**). *IgLON2* showed expression in the lateral habenula (LHb), the lateroposterior thalamic nucleus (LP), LD, VAL, VPM, VPL, and the mediodorsal thalamic nucleus (MD) at all ages analyzed (**D**–**F**). At E16 and P0, *IgLON2* could also be detected in the centromedial thalamic nucleus (CM), and expression in the PVT was observed at P15 (**D**–**F**). *IgLON3* was detected in the MHb, LHb, PVT, and MD (**G**–**I**). At P15, expression could also be detected in the LD, VPM, and VPL (**I**). At E16, *IgLON4* showed expression in the LHb, MHb, PVT, LP, LD, MD, CM, VPL, and the ventromedial thalamic nucleus (VM) (**J**). *IgLON4* was also observed in the majority of these nuclei at P0 and P15 (**K**, **L**). *IgLON5* mRNA expression was visible in the MHb at all ages analyzed. Low levels of expression were also detected in most thalamic nuclei. (**M**–**O**). All scale bars = 500 µm.

The thalamus is the central hub for sensory input, and largely acts as a relay station to the cerebral cortex^61^. The thalamic complex is composed of various thalamic nuclei, the habenula, and the hypothalamus^62^. The habenula is divided into medial (MHb) and lateral (LHb) regions, and functions mainly in relaying input received from the basal ganglia and forebrain to monoaminergic brain structures such as the ventral tegmental area^63^. The modality-specific nuclei in the thalamus are largely responsible for relaying sensory information to the cortex, while hypothalamic nuclei function in innate survival behaviors such as feeding and fighting^64–66^. *IgLON1* mRNA expression was detected in the MHb, the ventral anterolateral thalamic nucleus (VAL), and the laterodorsal thalamic nucleus (LD) at E16, and P15 (Fig. 5A, C). At P0, *IgLON1* could be detected in these nuclei as well as in the central posteromedial thalamic nucleus (VPM) and central medial thalamic nucleus (CM) (Fig. 5B). *IgLON2* showed expression at E16 and P0 in the LHb, MHb, LD, lateroposterior thalamic nucleus (LP), VAL, VPM, CM, the ventral posterolateral thalamic nucleus (VPL) and medial dorsal thalamic nucleus (MD) (Fig. 5D, E). The expression of *IgLON2* was maintained in these regions at P15 (Fig. 5F). At all ages analyzed, *IgLON3* mRNA was detected in the LHb, MHb, MD, and paraventricular thalamic nucleus (PVT) (Fig. 5G–I). At P0 and P15, some *IgLON3* expression was also detected in the LD, VPM, VPL, and LP (Fig. 5H, I). *IgLON4* mRNA expression was extensive at E16; mRNA was detected in the MHb, LHb, PVT, LP, LD, MD, CM, VPL, and ventromedial thalamic nucleus (VM) (Fig. 5J). *IgLON4* was also detected in all the above nuclei at ages P0 and P15 (Fig. 5K, L). *IgLON5* mRNA was observed in the MHb at all ages examined. Low levels of expression were also detected in the LHb and in most thalamic nuclei (Fig. 5M–O).

### *IgLON* expression in the hippocampus

**Figure 6:**
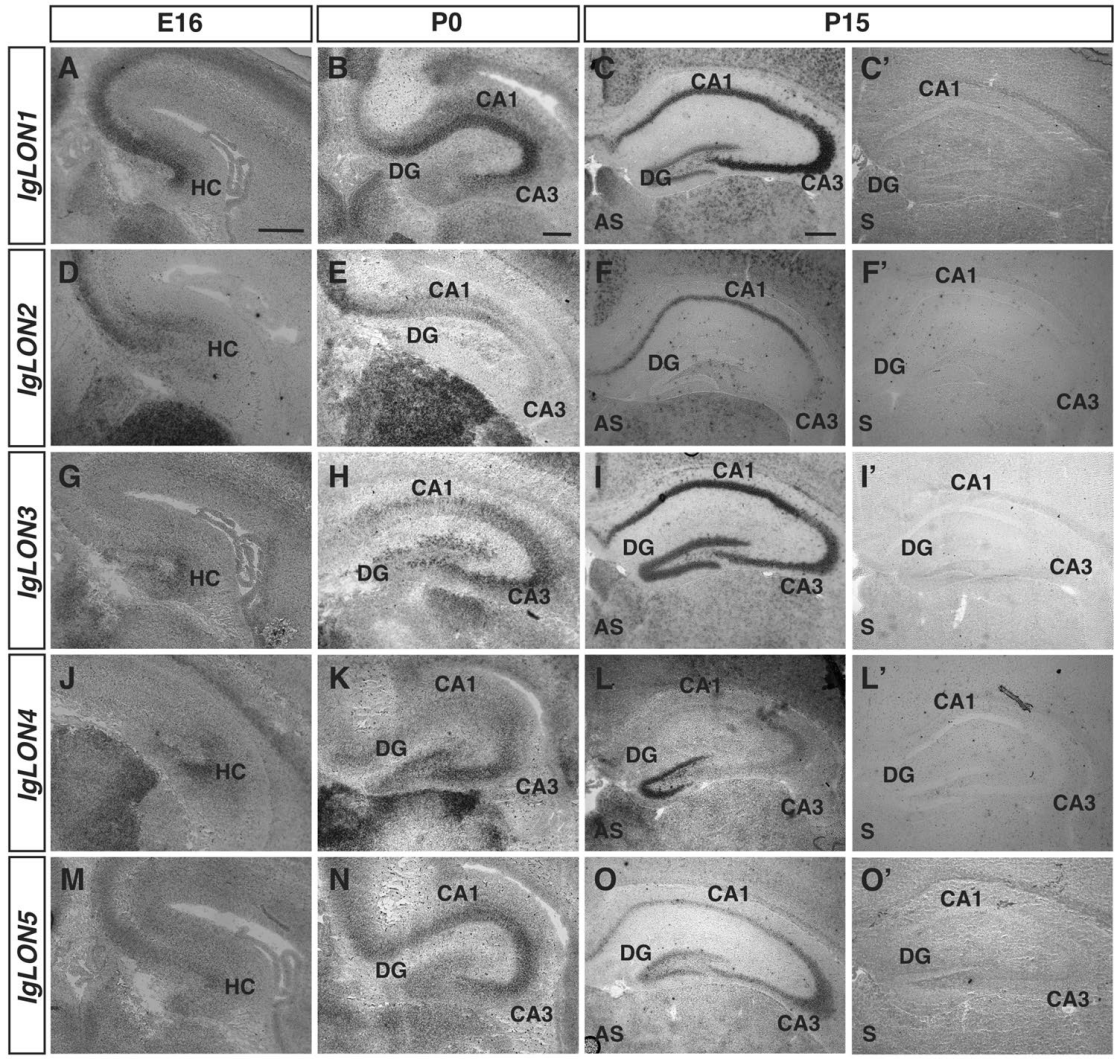
*IgLON* mRNA expression in the developing hippocampal region. In situ hybridization of coronal sections of the hippocampal region from E16, P0, and P15 mice with antisense (**A**–**O**) or sense (**C′**–**O′**) cRNA probes for *IgLON* transcripts. *IgLON1–5* are expressed in the developing hippocampal formation (HC) at E16 (**A**, **D**, **G**, **J**, **M**). *IgLON1* mRNA is observed in the CA1, CA3, and dentate gyrus (DG) of the hippocampus at P0 and P15 (**B**, **C**). *IgLON2* expression is restricted to the CA1 region of the hippocampus at P0 and P15 (**E**, **F**). *IgLON3* is uniformly expressed in both the CA1 and CA3 regions, as well as in the DG, during postnatal development (**H**, **I**), whereas *IgLON4* is expressed in the DG, CA3, and presumptive CA2 regions at P0 and P15 (**K**, **L**). *IgLON5* expression peaks at P0 in the CA1 and CA3 regions but dissipates by P15 (**N**, **O**). Scale bars = 250 µm (**A**, **B**, **D**, **E**, **G**, **H**, **J**, **K**, **M**, **N**), and 500 µm (**C**, **C′**, **F**, **F′**, **I**, **I′**, **L**, **L′**, **O**, **O′**).

The hippocampus functions in memory formation and spatial navigation. The hippocampal formation receives input from the entorhinal cortex to the granule cells of the dentate gyrus (DG). The DG cell axons project to CA3 pyramidal neurons as a bundle known as the mossy fiber pathway. The CA3 pyramidal cell axons in turn project to CA1 pyramidal neurons via the Schaffer collateral pathway^67,68^. Signals are then either relayed back to the entorhinal cortex or to the cerebral cortex. Our analysis of the expression patterns of *IgLONs* in the hippocampal formation revealed that all members of this family are expressed in various subregions. At E16, when it remains difficult to identify some subregions of the hippocampal formation, *IgLON1 to 5* mRNA could be detected (Fig. 6A, D, G, J, M). At P0, *IgLON1* was detected in the CA1-CA3 regions and in the DG (Fig. 6B). This expression was maintained at P15, although higher levels of expression was observed in the CA3 at that age (Fig. 6C). *IgLON2* was detected in the CA1 region at both P0 and P15 (Fig. 6E, F). *IgLON3* was expressed in the CA1–CA3 regions and in the DG at these ages (Fig. 6H, I). *IgLON4* was expressed in the DG, CA3, and presumptive CA2 region of the hippocampus at P0 and P15 (Fig. 6K, L). *IgLON5* was detected in the DG and in the CA1-CA3 regions at P0 and P15 (Fig. 6N, O). *IgLON2, IgLON3*, *IgLON4, and IgLON5* were also expressed at varying levels in the entorhinal cortex, which is a major source of inputs to the dentate gyrus of the hippocampus (Fig. S2B–E)^69^.

### *IgLON* expression in the cerebellum

**Figure 7:**
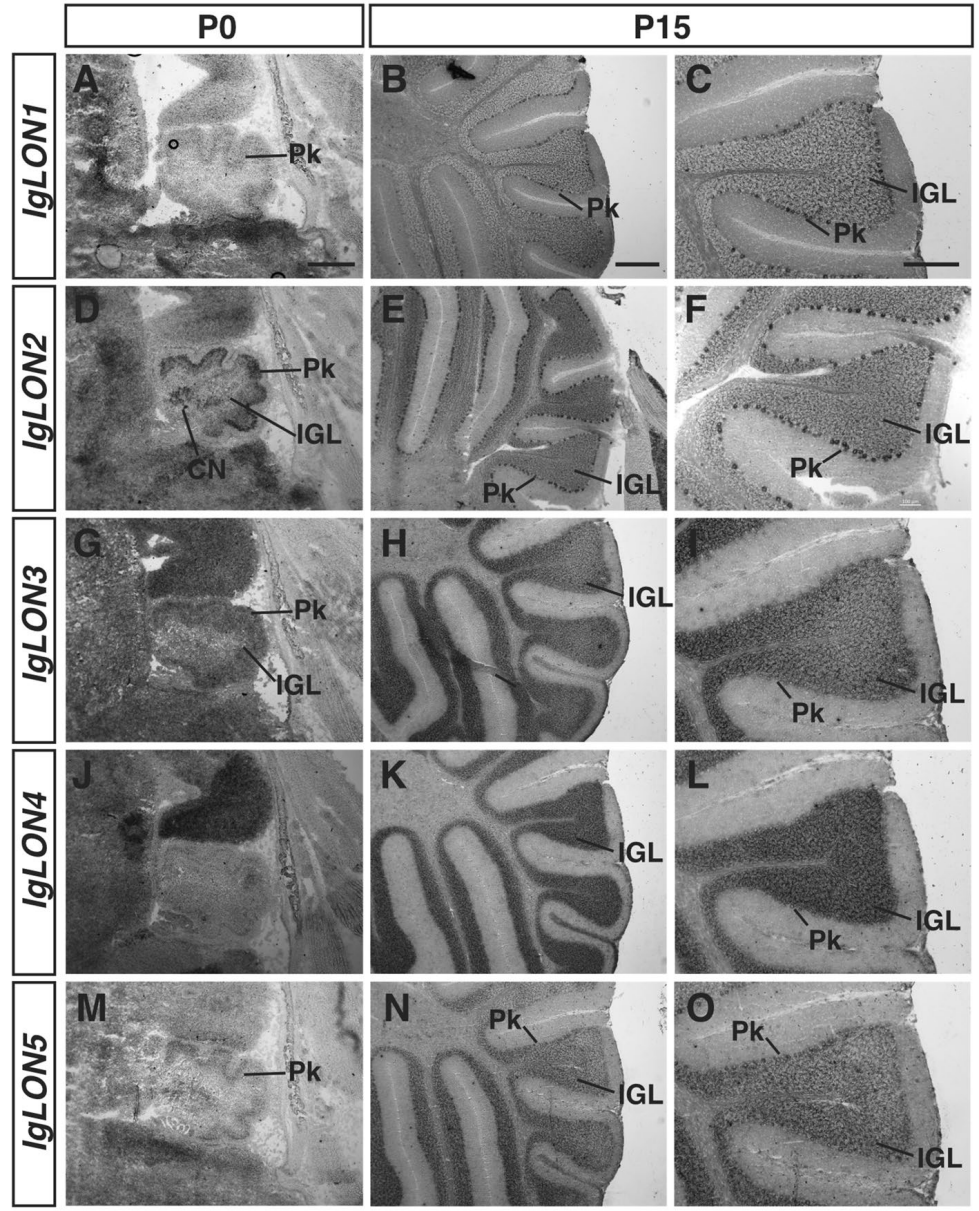
*IgLONs* mRNA expression in the cerebellum. In situ hybridization of sagittal sections from P0 and P15 mouse brains with antisense cRNA probes for *IgLON* transcripts. *IgLON1* was expressed at low levels in the Purkinje cell layer (Pk) at P0 and P15 (**A**–**C**). *IgLON2* showed expression in the Pk and in the internal granule layer (IGL) at P0 and P15 (**D**–**F**). *IgLON3* mRNA could be detected in the IGL and at low levels in the Pk at P0 and P15 (**G**–**I**). *IgLON4* showed weak expression in the Pk at P0 (**J**) but was restricted to the IGL by P15 (**K**, **L**). *IgLON5* mRNA expression was detected in the Pk layer at P0 and in both Pk and IGL layers at P15 (**M**–**O**). Scale bars = 200 µm (**A**, **C**, **D**, **F**, **G**, **I**, **J**, **L**, **M**, **O**) and 500 µm (**B**, **E**, **H**, **K**, **N**).

The cerebellum is important for controlling motor coordination^70^. The dense layer of excitatory granule cells receives input from various regions of the brain and project to Purkinje cells, which in turn mainly project to cerebellar nuclei. The different cellular and molecular layers of the developing cerebellum begin to be distinguishable around age P0 and are well-defined by age P15 (Fig. 7). *IgLON1* mRNA was detectable in the Purkinje layer (Pk) at P0 and P15 (Fig. 7A–C). *IgLON2* mRNA was detected in both the inner granular layer and in the Pk at P0 and P15 (Fig. 7D–F). *IgLON3* showed strong expression in the inner granular layer (IGL) and weak expression in the Pk (Fig. 7G–I). *IgLON4* mRNA could be detected at low levels in the Purkinje layer at P0 and P15, and its expression increased in the inner granular layer by P15 (Fig. 7J–L). *IgLON5* was expressed in the Pk at P0 and in the Pk and IGL at P15 (Fig. 7M–O).

### *IgLON* expression in the spinal cord and dorsal root ganglia

**Figure 8:**
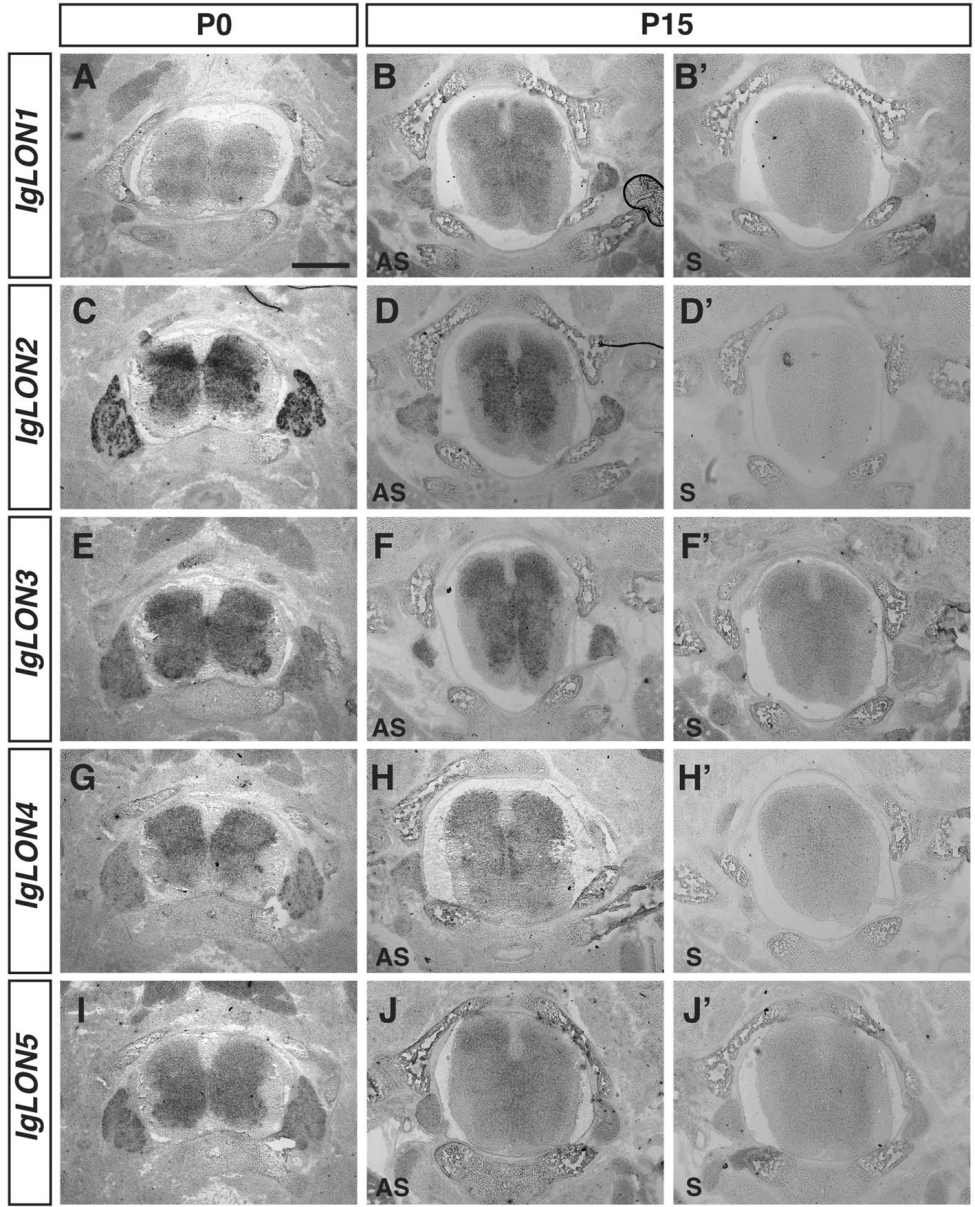
*IgLONs* mRNA expression during spinal cord development. In situ hybridization of coronal sections of the spinal cord from P0 and P15 mice with antisense (**A**–**J**) or sense (**B′**–**J′**) cRNA probes for *IgLON* transcripts. *IgLON1*, *IgLON2*, *IgLON3*, *IgLON4*, and *IgLON5* mRNAs are detected at various levels in the gray matter of the spinal cord at P0 and P15 (**A**–**J**). At P0, higher levels of expression of *IgLON2* and *IgLON4* are observed in the dorsal region of the spinal cord (**C**, **G**) while higher levels of *IgLON3* and *IgLON5* are detected in the ventral part of the spinal cord at P0 (I). All *IgLON* family members are expressed in sensory neurons within the dorsal root ganglia at P0 (**A**, **C**, **E**, **G**, **I**) while only *IgLON1*, *IgLON2*, and *IgLON3* mRNAs could be detected in these structures at P15 (**B**, **D**, **F**). Scale bar = 1 mm.

In the developing spinal cord, *IgLONs* are expressed in the grey matter and in the dorsal root ganglia (DRG) containing sensory neurons. *IgLON1, IgLON3, and IgLON5* showed widespread expression in the gray matter at P0 and P15, and in DRGs at both ages (Fig. 8A, B, E, F, I, J). While *IgLON3* is expressed throughout the grey matter, higher levels of expression are observed in the ventral part of the spinal cord containing motor neurons at E16 (Fig. 8E). *IgLON2* was also expressed in the grey matter but lower levels of expression were observed in the most dorsal lamina at P0 (Fig. 8D). *IgLON4* mRNA was enriched in the dorsal part of the spinal cord at both P0 and P15 (Fig. 8G, H) where sensory interneuron cell bodies are located (Fig. 8G, H). All *IgLON* family members were also detected in the cell bodies of DRG neurons. While *IgLON1*, *IgLON2*, *IgLON3*, and *IgLON5* appeared to be expressed in the majority of DRG neurons, *IgLON4* expression was detected in a subset of DRG neurons. Whether *IgLON4* is expressed in a subset of DRG neurons with a specific sensory modality remains to be established.

### *IgLON* expression in the developing eye

At E16, the retina is composed of a developing inner nuclear layer (INL), a retinal ganglion cell layer (GCL), and a proliferative neuroblastic cell layer (NBL) in which photoreceptors and horizontal cells will form (Kaufman, 1992). *IgLON1* and *IgLON4* mRNA was not detected in any eye structure at age E16 (Fig. S3A, D). *IgLON2* and *IgLON3* showed expression in the GCL, presumptive INL, and in the NBL (Fig. S3B, C). In addition, *IgLON3* was highly expressed in the lens of the eye (Fig. S3C). *IgLON5* only showed expression in the GCL and presumptive INL (Fig. S3E).

## Discussion

Cell adhesion molecules modulate a wide variety of processes during neuronal circuit development and maintenance. Although *IgLON* family members have been implicated in the regulation of some of these processes, including axonal growth and synapse formation, our understanding of their role in the development of neuronal circuitry in specific regions of the brain remains limited. To begin to gain insight into the roles of *IgLONs* in the development of specific regions of the nervous system, we have performed a detailed analysis of their patterns of expression in the murine nervous system. Our results show that *IgLON* family members are expressed in distinct patterns of expression and that their expression is temporally regulated during development. In multiple regions of the brain, several *IgLON* family members are expressed in a complementary fashion, suggesting a functional diversity or neuronal subtype-specific roles for the different *IgLONs*. Furthermore, in addition to being high during embryogenesis, *IgLON* expression is maintained postnatally in several structures analyzed, suggesting they also contribute to processes that take place later in development, including synapse formation and refinement.

During formation of olfactory glomerular maps, the differential expression of cell adhesion molecules on olfactory and vomeronasal receptor neurons contributes to the coalescence of subsets of axons expressing the same olfactory or vomeronasal receptors into specific glomeruli of the olfactory and accessory olfactory bulbs, respectively^71^. Although members of the Kirrel family of cell adhesion molecules have been shown to play an important role in this process, it is likely that additional cell adhesion molecules also modulate axonal coalescence during olfactory map formation. In *Drosophila*, the *IgLON* homologs, DIPs, are differentially expressed in olfactory receptor neurons and have been proposed to serve as tags to promote coalescence of axons expressing the same olfactory receptor during formation of glomeruli in the mushroom body^72,73^. Our examination of the patterns of expression of *IgLONs* in the mouse olfactory and vomeronasal epithelia revealed that most family members are highly expressed in olfactory and vomeronasal sensory neurons when axons are projecting and innervating their targets. However, we did not observe differential expression of *IgLON* family members among these neurons, suggesting they are unlikely to act as recognition tags for these projecting axons.

In contrast to the olfactory  epithelium, *IgLON* family members are differentially expressed in specific regions of several brain structures, such as the thalamus. It remains possible that the differential expression of *IgLONs* in the thalamus serves to modulate the fasciculation and segregation of different populations of projecting axons. In keeping with this possibility, proper fasciculation of dopaminergic afferent fibers and guidance to the lateral habenula requires IgLON3 expression on lateral habenula efferent projections^33^. *IgLONs* are also differentially expressed in regions of the hippocampus. While *IgLON2* expression appears restricted to the CA1–CA2 region, *IgLON1* is more highly expressed in the CA3 region than in the CA1–CA2 region, and *IgLON4* is more highly expressed in the dentate gyrus. In vitro, expression of IgLON1 or IgLON3 in hippocampal neurons increased synapse numbers while expression of IgLON4 reduced synapse numbers^37^. The differential expression of *IgLONs* in the hippocampus may therefore contribute to synapse specificity by promoting or inhibiting synapse formation between specific populations of neurons in the hippocampus. The expression of *IgLONs* may also regulate axonal growth in this system as cell surface cleavage of IgLON4 in cultures of primary neurons can promote neurite outgrowth^30,74,75^.

The patterns of expression we observe mostly correlate with previously observed expression profiles in specific regions of the brain described in the literature^17,21,22,32,51,52,76,77^. However, while we observed expression of *IgLON3/Lsamp* in the developing cortex at E16, we did not detect its expression in the ventricular zone as previously reported for *Lsamp 1b*^20^. In contrast to our in situ hybridization approach, Philips et al. examined the expression of *Lsamp 1b* using a very sensitive Xgal enzymatic reaction on brain sections from mice carrying an insertion of a *LacZ* gene cassette in-frame with the *Lsamp* 1b promoter. It is therefore likely that our inability to detect *IgLON3* expression in the ventricular zone is due to a lower sensitivity of the in situ hybridization approach we have used. Alternatively, it is possible that the *Lsamp 1b* transcript is expressed in the ventricle at earlier time-points during development and downregulated by E16, but that residual β-galactosidase protein is still present at that age in the *Lsamp 1b-lacZ* mouse, leading to an Xgal-positive signal in this structure.

In conclusion, our survey of *IgLON* expression shows that *IgLONs* are widely expressed in the developing nervous system, with partially overlapping, yet distinct, patterns of expression. These analyses will permit the future development of in vivo assays to address the function of these proteins in the development and maintenance of specific regions of the nervous system using available and newly developed genetically-modified mouse models.

## Methods

### Animals and ethics

E16 mouse embryos were obtained from timed-pregnant females of the CD1 strain purchased from Charles River. P0 and P15 CD1 mouse pups were obtained from Charles River. The date of vaginal plug was considered E0, and the day of birth was considered P0. Heads and embryos were cryoprotected with Tissue-Tek O.C.T. compound (Miles, Elkhart,IN) and flash-frozen in dry ice cooled 2-methylbutane. Tissue was stored at − 80 °C until cryosectioning. All animal procedures used were approved by the Neuro Animal Care Committee and McGill University, in accordance with the guidelines of the Canadian Council on Animal Care. Data and methods are reported in accordance with the ARRIVE guidelines^78^.

### Riboprobe synthesis

For each *IgLON* member, digoxigenin-labeled cRNA probes with either antisense or sense orientation were prepared by in vitro transcription using DIG labelling mix (Sigma,USA). Probe sequences were PCR-amplified from cDNAs prepared from mouse brain cDNA using primers designed based on sequences from the Allen Brain Atlas and Gene Paint Database (see Supplemental Material for probe sequences) and cloned into a pBluescript vector. cRNA probes were designed to detect expression of both the 1a and 1b transcripts for *IgLON1*, *IgLON2*, and *IgLON3*. Following in vitro transcription, the riboprobes were partially hydrolyzed in a 10 mM DTT and 200 mM NaHCO_3_/Na2CO_3_ solution (pH 11) for 25 min at 60 °C^79^. Hydrolysis was stopped by addition of 100 mM acetic acid, and cRNA probes were precipitated by addition of 1/10th volume 4 M LiCl and ethanol. Precipitated cRNA fragments were resuspended in diethylpyrocarbonate (DEPC)-treated water and stored at − 80 °C.

### In situ hybridization

In situ hybridization was performed as previously described^80^. 20 µm thick sections of fresh-frozen heads or embryos were thaw-mounted onto Fisherbrand Superfrost Plus microscope slides (Fisher Scientific, Hampton, NH) and allowed to dry for 1 h. Sections were incubated in diethyl pyrocarbonate (DEPC)-treated 4% paraformaldehyde in 0.1 M phosphate-buffered isotonic saline (PBS; pH 7.4) for 20 min and washed 3 times in 1 × PBS for 5 min each, followed by acetylation in 0.25% acetic anhydride in 1% triethanolamine solution for 10 min. Sections were washed in 1X PBS and 1X standard saline citrate (SSC; pH 7.4) and pre-hybridized in hybridization solution (50% formamide, 5 × Denhardt's solution, 5 × SSC, 200 µg/ml baker’s yeast tRNA) in a humidified chamber at 60 °C for 2 h to overnight. Slides were then incubated overnight at 60 °C in hybridization solution containing approximately 100 ng/ml of *IgLON* cRNA probes.

The following day, slides were washed in 5 × SSC at 60 °C for 5 min and 2 × SSC at 60 °C for 1 min. The slides were then incubated in 0.2 × SSC in 50% formamide solution at 60 °C for 30 min, followed by a 5-min wash in Tris-buffered saline (TBS; 100 mM Tris-HC1, pH 7.5, 150 mM NaCl) and a 1-h block in 1% blocking reagent (Sigma) in TBS. After a 5-min wash in TBS, slides were incubated with anti-digoxigenin Fab fragments conjugated to alkaline phosphatase (1:3000) for 3 h, washed in TBS, and subjected to a color reaction overnight at room temperature. Slides were coverslip mounted with Mowiol 4-88 (Calbiochem, San Diego, CA) and sections examined on a Zeiss Axio imager upright microscope. Each in situ hybridization experiment was repeated a minimum of three times on a minimum of three different samples to confirm the patterns of expression observed.

## Supplementary Information

Supplementary Information 1.
