# Supplementary material for: Spatiotemporal expression of IgLON family members in the developing mouse nervous system
Source: Sci Rep. 2021 Oct 1;11:19536. doi: 10.1038/s41598-021-97768-5 (PMC8486791; doi:10.1038/s41598-021-97768-5)
Supplement: Supplementary file 1 — Supplementary Information 1. [file 41598_2021_97768_MOESM1_ESM.pdf]

Supplementary Information

Spatiotemporal expression of IgLON family members in the developing mouse nervous system.

Sydney Fearnley, Reesha Raja, and Jean-François Cloutier.

IgLON cRNA probe sequences (primer sequences are underlined)

IgLON1:

TTCTAAAACTTCTCCCCTTCCCAAACCAATGCTAATTTGTTCTTCCACCAAAGTATGC
CAATGAAAGTGCTAGTGTTCTGCCTCTGGCAAAGCCTGTTTTTTGAATAGTTTAATGT
CAAGTGCCTGATACAGTCATCTGCAAGTTTAATCAAGAGTGTTTGGATTTTCTTTTTT
TGTTCTCATTGGTTAGGTTGGAGACATAGTAGATTAGTTGTCAAAACATATACAGCT
CTGACACAGAGAGCTAGGTATGTGGCTCTTCTGCTGTGGGCGAAGCTGTGTTCAACA
GATGGAAATGGACATCTGTATGTCACCAAGATGGCTCATGCCTGTCCTTACACTGCT
TTGGGCTGTTGTTCACAGGTTGGGAAGTTAGTTTTCAAAATATGGTCATAGGTTTGG
TTTGGAATTCTAGGACCTTCATAACTGAGGCTGCATTTTAATGATCTCTTTCTATATC
CATCTGGTCACATTGTCCTCAGCAAGAAGGAATAGCAAACCTGCCTACAATAGGAA
AAATATCAAAAGAGCAGAGCCCCACCTTCCCCAAGTGGACACTGGATCCCAGAGAG
TTTATCACAGGCACTGGATAAAGAAAAGTTGGAAATTTAATACAAATGATTTGATTG
ATACTTCCAGGGACAAAGAACACATGCTCTTC

IgLON2:

ACCTCCAGGGTCCACCTCATTGTACAAGTATCTCCCAAAATTGTAGAGATTTCTTCA
GATATCTCCATTAATGAAGGGAACAACATCAGCCTCACTTGCATAGCCACAGGTAG
ACCGGAGCCTACAGTAACCTGGAGACATATTTCTCCCAAGGCCGTTGGCTTTGTGAG
TGAGGATGAGTACCTGGAGATCCAGGGCATCACTCGGGAACAGTCAGGCGAGTACG
AGTGCAGCGCCTCCAACGACGTGGCGGCACCAGTGGTACGAAGAGTGAAGGTCACC
GTGAACTATCCACCATACATCTCAGAAGCTAAGGGCACAGGTGTCCCCGTGGGGCA
GAAGGGGACTCTGCAGTGTGAAGCTTCCGCAGTCCCTTCAGCAGAATTTCAATGGTT
CAAGGATGACAAAAGACTGGTCGAAGGAAAGAAGGGAGTCAAAGTGGAAAACAGA
CCTTTCCTTTCAAAACTCACCTTTTTCAACGTCTCTGAACATGACTATGGGAACTACA
CATGTGTGGCCTCCAACAAGCTGGGTCACACCAACGCCAGCATCATGCTATTTGGTC
CCGGTGCTGTCAGTGAGGTCAACAATGGGACATCAAGGAGGGCAGGCTGCATTTGG
CTCCTCCCTCTTCTGGTCTTACACCTGCTCCTCAAATTTTGATGTGAGTGCCCCTTCC
CTGCTGGGGAGAGCTGCTGCCACCGCATCTCAATACAACAGCACTGCAAAATGAAG
CAACAAGTCAGAATCAAATGAAATTCCGAGAATCACAGCCAATGAGACAGAAATTC
GAGGGAGGGGGACAAAGCATACTGTGGTAAAGGGGAAAAAAAGGTTTAAGAAAAG
GAAATTTGGAAATTGCCTTGCAGATATTTCGGTACCGCTGAGTTTTCTTTCTTTTCCC
AAGTGGGAAGAAGGCACACCTAGCTTGGACCCACCCACAAGCTGCACT

IgLON3:

CTCAGTTCAGACACAGCATGAGCCCAAGACCTCCCAAGTTTACTTGATCGTACAAGT
TCCACCAAAGATCTCCAACATCTCCTCGGATGTCACTGTGAATGAGGGCAGCAATGT
AACCCTGGTCTGCATGGCCAATGGGCGCCCTGAACCTGTTATCACCTGGAGACACCT
TACACCACTGGAAGAGAATTTGAAGGAGAAGAAGAATATCTGGAGATCCTAGGCAT
CACCAGGGAACAGTCAGGCAAATATGAGTGCAAGGCTGCCAACGAGGTCTCCTCGG
CGGATGTCAAACAAGTCAAGGTCACTGTGAACTATCCACCCACTATCACGGAGTCG
AAGAGCAACGAAGCCACCACAGGACGACAAGCTTCCCTCAAATGTGAAGCCTCAGC
GGTGCCTGCACCTGACTTTGAGTGGTACCGGGATGACACCAGGATAAACAGTGCAA
ATGGCCTTGAGATTAAGAGCACTGAGGGCCAGTCCTCCCT**G**ACGGTGACCAATGTC
ACTG

IgLON4:

ACGGACGATGCAGGTTCATCTCACTGTGCAAGTTCCACCGAAAATATATGACATCTC
AAATGACATGACCATCAATGAAGGAACCAACGTCACCCTTACTTGTTTGGCCACTGG
GAAGCCAGAGCCCGCCATTTCCTGGAGGCATATCTCCCCATCAGCAAAACCATTTGA
AAATGGACAATATTTGGACATTTATGGAATTACAAGAGACCAGGCTGGGGAGTACG
AATGCAGTGCAGAGAACGATGTATCATTCCCAGATGTGAAGAAAGTGAGAGTGGTC
GTGAACTTTGCGCCTACAATTCAGGAAATTAAATCTGGCACAGTGACCCCTGGACGC
AGTGGACTGATAAGATGTGAGGGTGCAGGTGTGCCGCCGCCAGCCTTCGAGTGGTA
CAAAGGAGAGAAGAGACTCTTCAATGGCCAACAAGGAATTATCATTCAGAATTTTA
GCACAAGATCCATCCTCACAGTGACCAACGTGACACAGGAGCACTTCGGCAACTAT
ACTTGTGTGGCTGCCAACAAGTTGGGCACAAC

IgLON5:

CTCCGAGACGGTTTCACCTCAGAGGGTGAGATCCTGGAAATCTCAGACATCCAGCGGGGCC
AGGCCGGGGAATACGAATGCGTTACTCACAACGGAGTGAACTCGGCGCCCGACAGCCGCCG
TGTGCTGGTCACAGTGAATTATCCCCCGACCATCACAGATGTGACAAGCGCGCGCACCGCCC
TGGGCCGGGCTGCCCTCCTGCGCTGTGAAGCTATGGCTGTGCCGCCGGCAGATTTCCAGTGG
TACAAGGATGACAGGCTGCTAAGCAGCGGTTCTGCAGAGGGCCTCAAGGTGCAGACCGAGC
GCACCCGCTCTATGCTTCTCTTCGCCAACGTGAGCGCCAGGCACTATGGCAACTACACCTGC
CGTGCGGCCAACCGGCTCGGAGCGTCCAGCGCCTCCATGCGGCTCCTGCCCCAGGATCCCTG
GAGAACTCAGCTCCAAGGCCTCCAGGGCCCCTGACCCTCCTCTCTGCCCTGAGCTGGCTGTG
GTGGAGAATGTAGACACCATTCAGTACAGCGCA

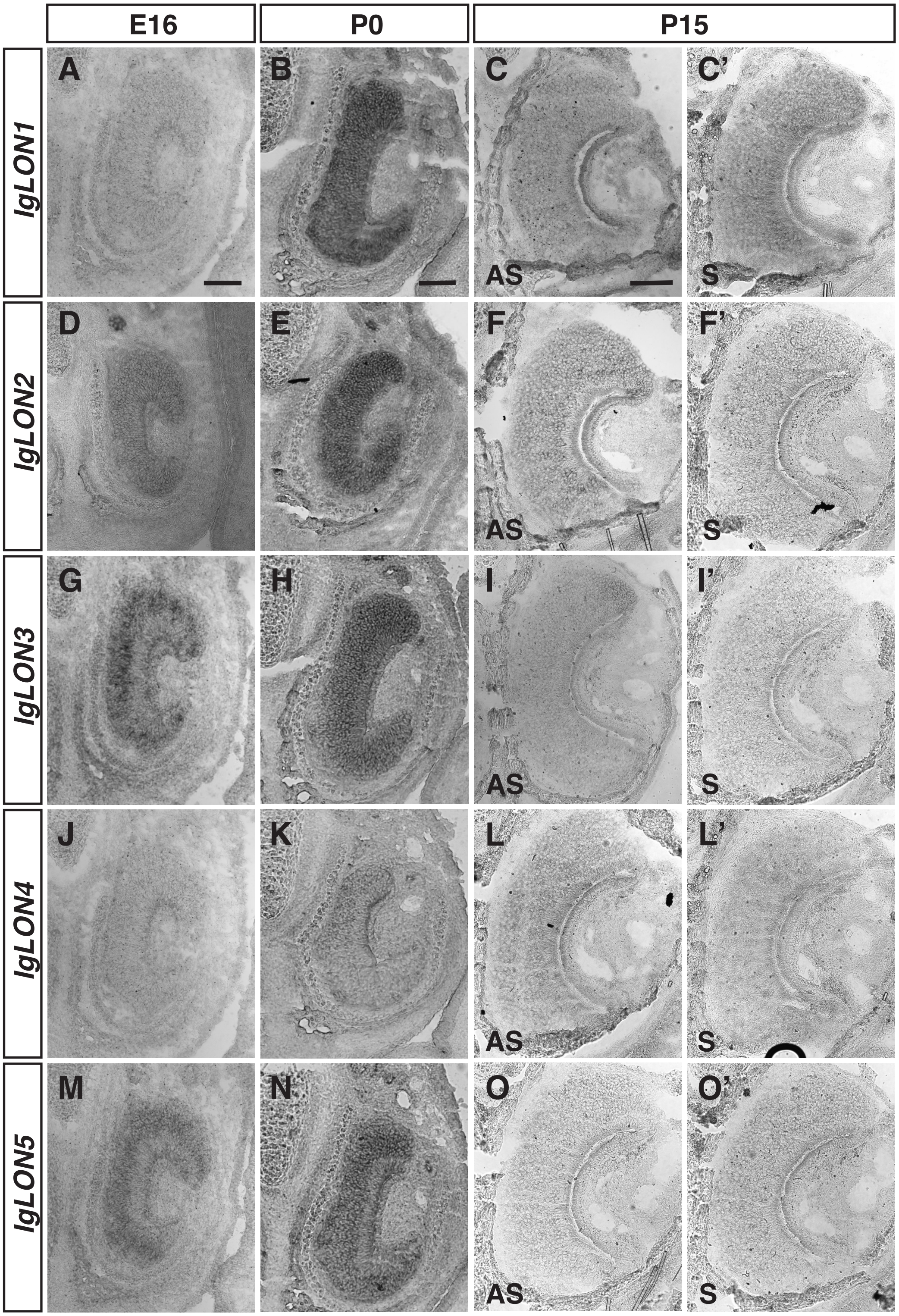

Supplemental Figure 1. *IgLONs* mRNA expression in the developing vomeronasal organ (VNO).

In situ hybridization of coronal sections of OE from E16 and postnatal days P0 and P15 with antisense (A-O) or sense (C', F', I', L', O') cRNA probes for *IgLON* transcripts. *IgLON2, IgLON3,* and *IgLON5* mRNAs are detected in the VNO at E16 (D, G, M), while *IgLON1* and *IgLON4* were not expressed at that age (A, J). By P0, high levels of I*gLON1, IgLON2, IgLON3,* and *IgLON5* mRNAs were observed in the VNO (B, E, H, N). Expression of IgLON family members was downregulated beyond detectable levels in the VNO by P15 (C, F, I, L, O). Scale bars = 100 µm.

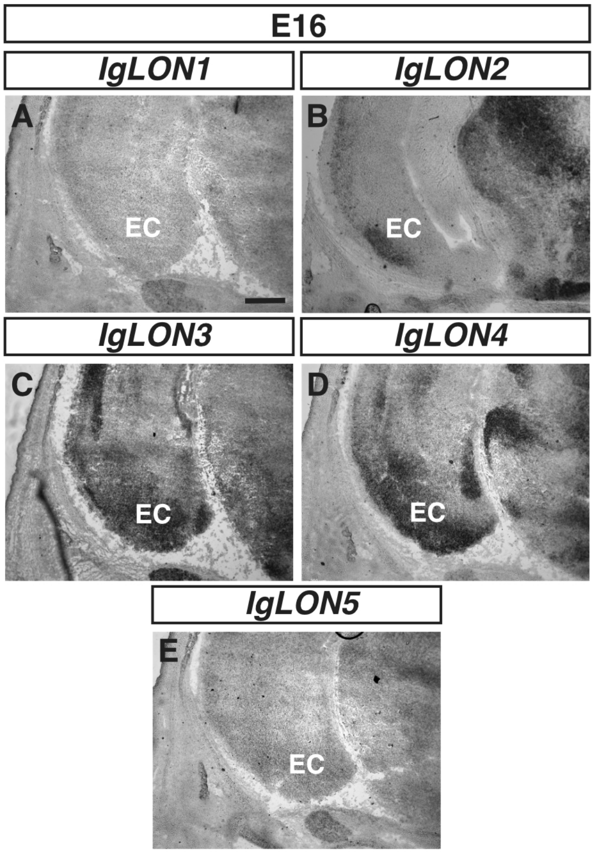

Supplemental Figure 2 *IgLONs* mRNA expression in the entorhinal cortex.
In situ hybridization of coronal sections of entorhinal cortex from E16 emrbyos with antisense cRNA probes for *IgLON* transcripts. *IgLON2*, *IgLON3, IgLON4,* and IgLON5 were detected (C, D). Scale bar = 500 μm.

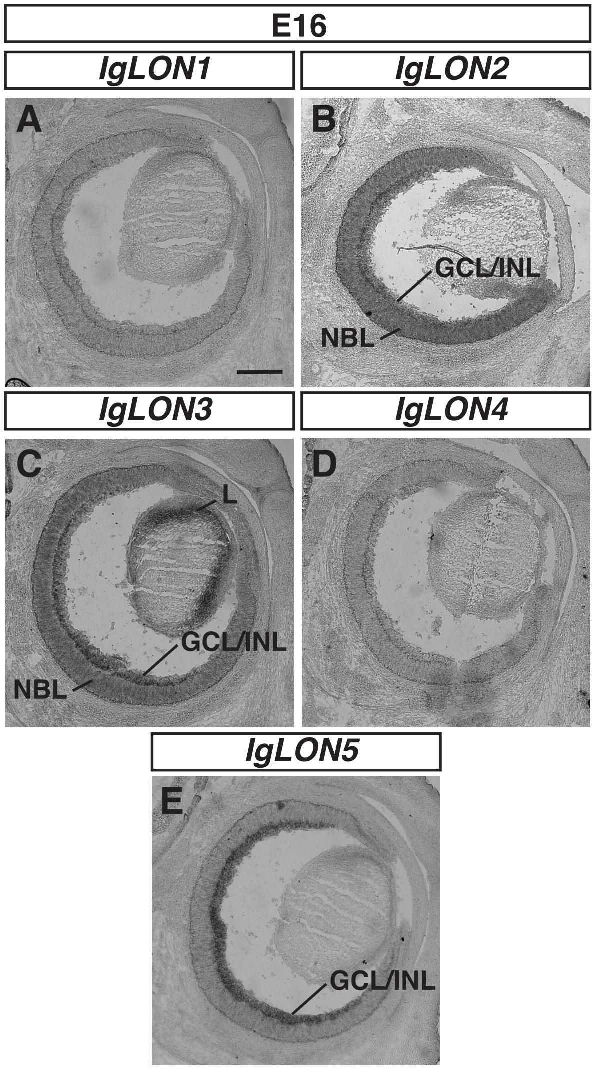

Supplemental Figure 3. *IgLONs* mRNA expression in the developing eye.
In situ hybridization of coronal sections of the eye from E16 embryos with antisense cRNA probes for *IgLON* genes. *IgLON2* and *IgLON3* are expressed in the ganglion cell layer (GCL), presumptive inner nuclear layer (INL), and in the neuroblastic cell layer (NBL) (B, C). *IgLON3* is also expressed in the lens of the eye (L) (C). *IgLON5* expression is restricted to the GCL and presumptive INL (E) while *IgLON1* and *IgLON4* mRNAs are not detected in the eye (A, D). Scale bar = 200 µm.
